# Standardizing the Conception of Cardiovascular Depression

**Published:** 1920-03

**Authors:** 


### STANDARDIZING THE CONCEPTION OF CARDIO-VASCULAR DEPRESSION

Speaking before a joint meeting of the American Association of Obstetricians and Gynecologists and the Interstate Association of Anesthetists at Cincinnati, September 15, 1919, Drs. Charles W. Moots and E. I. McKesson emphasized the fact that cardio-vascular depression being the outstanding symptom of the condition known as shock, it is reasonable to start with the proposition that whatever means enable us to determine the very beginning of this condition is of the greatest importance. These authorities hold that:

"When a cardio-vascular system is reacting normally, an increased pulse rate is accompanied by an increased systolic and diastolic blood pressure, and *vice versa*. The pulse pressure is roughly half as great as the diastolic pressure and is the most direct evidence we have of the amplitude of the heart contraction, the best evidence of effective blood movement. In normal sleep, the pulse rate and blood pressures are lowered, but their normal relationships are maintained; so are they in an ideal anesthesia.

"But during surgical operation, so many factors enter to disturb the normal reaction of the circulation that we may have many combinations, with almost never a true stimulation, but very frequently a depression of the circulatory system. The changes occur so frequently, with sometimes disastrous and sometimes innocent results, that it is most desirable to be able to differentiate between them and to anticipate their onset.

### BLOOD PRESSURE RULES

"There is no form of anesthesia, there is no age of patient, there is no type of operation in which one expects to see an elevation of blood pressures during the operation. Our fears are from low blood pressures, rapid pulse rate and heart fatigue.

"Circulatory depression or decompensation is best divided for surgical operation into three degrees:

"1. *Safe.* Ten to 15 per cent. increase on pulse rate without change in pressure. Ten to 15 per cent. decrease in blood pressures without change in pulse rate.

"2. *Dangerous.* Fifteen to 25 per cent. increase in pulse rate with 15 to 25 per cent. decrease in blood pressures.

"3. *Fatal.* Progressively increasing pulse rate above 100 with progressively falling blood pressures of 80 or less systolic and 20 or less pulse pressure, for more than 20 minutes.

"The first degree is never fatal, but may gradually merge into the second degree. The second degree, beginning shock, may be regarded as dangerous in the sense that it exhausts the heart and disarms it for defense against continued low blood pressures.

"The third degree is always dangerous to the life of the patient. A vicious circle is established consisting of the low blood pressure, the reduced heart nourishment, which in turn still further reduces the blood pressure, and so on progressively. This usually develops within 20 minutes after the third degree depression occurs, and when once well established proves fatal at once or at most within three days. The time in which shock proves fatal depends upon the cardiac muscle reserve and the effectiveness of the treatment employed. Third degree depression may be present in a patient without the usual alarming signs, but after the vicious circle becomes established, evidences of shock become well marked.

### VALUE OF BLOOD PRESSURE READINGS

"With the palpating finger, no matter how skilled, one can not determine all the characteristics of the pulse or the pulse pressures with sufficient accuracy to be of much prognostic value as to the onset and degree of circulatory depression during a surgical operation.

"Blood pressures and pulse determinations every few minutes during all of the more serious operations, as well as in many of the so-called minor cases, are a part of the duties of every anesthetist. The information regarding the patient's fitness for the operation, his reaction to certain procedures and the immediate prognosis can be gained in no other way with the same degree of accuracy.

"The procedure is made convenient and easy by fastening the blood pressure cuff to the right arm and snugly binding the stethoscope below it with elastic webbing.

Readings can then be made at will without disturbing sterile sheets and without losing the continuity of anesthesia.

PRESERVING MUSCLE TONE

"A suitable graphic chart is preferable as a record because the tendencies of the circulation are readily compared from time to time and because the prognosis based upon these tendencies and the character of operative work to follow can be more accurately made.

"Where nitrous oxid-oxygen was available in skilled hands the war has corroborated our previous observations that this form of narcosis is one of the best shock prophylactics we have.

"It is not remarkable that nitrous oxid-oxygen should be safer in shock and in preventing shock than other anesthetics when one recalls the fact that muscle can not be paralyzed with it.

"The greatest responsibility of the anesthetist is to avoid relative overdosing of the patient in an effort to please the surgeon who may be demanding a flabby musculature.

"The relaxation is not confined to striated muscles of the abdomen and extremities, but extends to the striated muscle of the heart. The effect is at once reflected by the pulse pressure and if pushed too far the diastolic pressure is also decreased, showing the action upon smooth muscle as well.

"The clinical study of blood pressure has convinced us that the final factor in shock is muscular exhaustion or an interference with muscular action. One thing is most apparent: the average patient having been profoundly anesthetized for extreme relaxation, is half shocked; a second degree depression, and it often takes but little trauma to complete the picture of third degree depression."

In this connection it is interesting to report that all the members of the Toledo Society of Anesthetists have adopted this standardized conception of cardio-vascular depression and are using it graphically on their charts. Their records when compiled should develop some valuable and original information.

